# Eye on the Ball: Table Tennis as a Pro-Health Form of Leisure-Time Physical Activity

**DOI:** 10.3390/ijerph15040738

**Published:** 2018-04-12

**Authors:** Elżbieta Biernat, Sonia Buchholtz, Justyna Krzepota

**Affiliations:** 1 Department of Tourism, Collegium of World Economy, Warsaw School of Economics, Al. Niepodległości 162, 02-554 Warsaw, Poland; 2 Department of Economics I, Collegium of Economic Analysis, Warsaw School of Economics, Al. Niepodległości 162, 02-554 Warsaw, Poland; sonia.buchholtz@sgh.waw.pl; 3 Department of Physical Culture and Health Promotion, University of Szczecin, Al. Piastów 40b, blok 6, 71-065 Szczecin, Poland; justyna.krzepota@usz.edu.pl

**Keywords:** table tennis, leisure-time physical activity, amateur sport, Poland, binary logistic regression

## Abstract

*Background:* The article is devoted to an analysis of leisure-time (amateur) table tennis in Poland, its practitioners and the regularities of their activity. *Methods:* The study examined 12,406 persons in 4689 households (representative for the population). We used binary logistic regression and descriptive statistics in order to identify the patterns and determinants of table-tennis practice in Poland. *Results:* Table tennis is practised by 2.8% of population, and by 6.6% of physically active Poles. Among adults it is predominantly an occasional recreational game, not performed as a sport per se. Among children, it is often the part of physical education (PE) classes. Statistically significant predictors of contact with table tennis are: gender, age, income, place of residence, children in the household and being a student. *Conclusions:* Due to the undeniable benefits of table tennis (health, pleasure, personal and social development), the sport is recommended for use as a tool in increasing the (overall low) physical activity of Poles. Its popularization requires promotion in the media (as a health-oriented activity) and using various channels, including public places, the workplace (as part of corporate social responsibility) and physical education classes at school.

## 1. Introduction

Sport is not a favourite leisure-time activity for adult Poles. Even though they declare insufficient time for relaxation (50%), they rarely turn to sport (7%). Non-working days are spent on watching TV (52%) or on passive relaxation (27%), but seldom on sports activities (11%) [1]. According to the Eurobarometer survey, 52% Poles admit to neither exercising nor playing sports at all (EU 28 mean—42%, below 15% among leaders), and another 18% rarely do so [2]. Consequently, according to the World Health Organization (WHO) framework [3], 41% of adult Poles has insufficient physical activity to maintain health [4], of which 24% exhibits a completely sedentary lifestyle. For groups at risk (poorly educated, farmers, non-working individuals) the percentage of insufficiently active exceeds 5 0%. Among the young a similar percentage (43%) was found [5], while 22% performed no activity at all [6]. It would seem that insufficient physical activity—with its financial and non-financial consequences [7,8,9] (Lee, et al., 2012)—poses a huge challenge for public health policy in Poland.

In contrast to physical activity, passive watching sport on TV has many supporters. 70% of adult Poles were interested in the Rio Olympics [10], of whom 1 in 10 declared the intention to follow table tennis competitions—far less than the most popular disciplines (handball, athletics, indoor volleyball, with over 40% interest), but outperforming several other disciplines (such as badminton, judo, or sports gymnastics). Such a poor result for table tennis is very surprising for two reasons. Firstly, Poland has a long history of successes in this discipline, due to native and naturalised players, both male and female: Andrzej Grubba, Leszek Kucharski (1980s), Lucjan Błaszczyk (1990s–2000s), Natalia Partyka, Xu Jie (2000s–2010s), to name only a few [11]. Secondly, for many years table tennis equipment has been a basic feature of public cultural, educational, and recreational public institutions, such as holiday resorts, student dormitories, playgrounds etc. [12].

Studies of table tennis are multifaceted. They have mostly focused on elite character of this sport and analysed physiological performance of athletes (energy expenditure during a training session or a match) [13,14] and the resultant practical implication for coaches [13]. They have identified determinants of talent [15,16] and level of achievement in the sport [17]. They have also explored the development of modern technologies, which has a fundamental impact on being successful, including rule changes [18] change of the ball size [19] change ball material [20] improvement of the racket and rubber technologies [21] and game temporal structure [22]. They have also examined the effect of intellectual disability on tactical [23] and technical proficiency [24] of table tennis players, which is connected with the second group of studies, concerning the effect of the sport on human body functions. Researchers who have explored these problems emphasized benefits of playing table tennis: better hand-eye coordination, improvement of reflexes, balance and coordination, brain stimulation and development of mental acuity, burning calories and as a social outlet [25]. They suggested that regular training can help maintain mental capacity and prevent or delay senile dementia [26] and (by moulding motor skills social behaviour) can be a great therapeutic agent for children with attention deficit hyperactivity disorder (ADHD) [27], autism [28] and mild mental disabilities [29]. Since table tennis is numbered among one of the fastest ball games in the world [13,15], a major part of studies have focused on strategies of visual search [30]. Other studies have shown that table tennis can also improve the dynamic visual acuity (DVA) of the general population [31], the biomotor capacities of children in general [32] and static balance of deaf children [33]. It is easy on the joints and rarely becomes traumatic [25]. Thus, it is perceived as a Leisure Time Physical Activity (LTPA) for the whole population spectrum. 

In this context, one can ask the reasonable question whether the desired increase in LTPA can come through table tennis. This measure should be taken under advisement. There is strong evidence that leisure-time table tennis training, with the potential for permanent implementation, is associated with beneficial effects on body composition and lipid profile in older male adults [34], that table tennis participants have a significantly higher life satisfaction and physical self-concept than non-exercisers [35], and that the table tennis exerted a greater influence on cognitive function than other types of exercise did [36]. Despite these documented health benefits, studies on the popularity of table tennis as a form that improves LTPA are scarce and relatively unavailable to general public [37]. According to authors, very few of them have demonstrated the correlation of socio-economic and demographic factors with leisure-time involvement in table tennis [38,39,40,41]. 

Bearing this context in mind, the aim of this study was to analyse participation of adult Poles in table tennis and factors that determine involvement in the sport in leisure time. We would like to identify people who tend to choose such an activity and what the patterns of the involvement in table tennis are among Poles. In our opinion, this knowledge can be critical to planning the strategies for promotion and popularization of the sport (at both local and national levels). It should become a basis for promoting and restoring interest in this sport, and as a result, may support increasing leisure-time physical activity in Poland. 

## 2. Materials and Methods

In this article we make use of the survey ‘Participation of Poles in sport and physical recreation 2012’ (‘Uczestnictwo Polaków w sporcie i rekreacji ruchowej w 2012 roku’). The survey was commissioned, designed and carried out by the Central Statistical Office of Poland (CSO, Główny Urząd Statystyczny). Its ID number is DS-52. This paper-and-pen interview (PAPI) was originally designed to analyse the participation in sport and physical recreation as an indicator of the quality of life of Poles. The primary objectives of this survey included the identification of: (1) preferred sport and physical recreation activities, (2) participation of individuals in them, (3) the level of skills, (4) reasons for activity and obstacles leading to inactivity, (5) household sports equipment and estimates of sport- and recreation-related expenditure. The survey analysed the abovementioned issues over the period of one year (October 2011–September 2012) and is representative for the entire population of Poland. 

In order to capture both household and individual perspectives, two questionnaires were used. The former (DS-52 G) covered the characteristics of the household, sports equipment and expenditures, physical condition of its members, as well as barriers to sport and physical recreation (if an insufficient level was declared). The latter, individual questionnaire (DS-52 I) was filled in by all household members, except for children aged 5–9, whose answers were provided by parents, and children below 5, for whom neither sport nor physical recreation was measured. Individual questionnaire included: skills, participation in training, description of the practice of selected disciplines, and motives for performing sport or physical recreation. Thirty-one (groups of) disciplines were distinguished, of which one was table tennis. Moreover, a wide range of demographic and socio-economic characteristics was captured: gender, age, marital status, education, income and its source, place of residence, size and structure of the household, socio-economic status, and labour market status.

It was possible to gather this level of detail due to the fact that ‘Participation…’ is a spinoff survey of the Polish ‘Household Budget Survey’ (HBS, ‘Badanie Budżetów Gospodarstw Domowych’). Originally, HBS used two sub-samples: one drawn in 2011 for 2012–2013 and another one drawn in 2010 for 2011–2012. The ‘Participation…’ survey uses only the latter sub-sample, namely the part surveyed for the last time in the third quarter of 2012. The sampling procedure in both surveys is based on a two-stage stratified sampling scheme with various probabilities of selection at stage one. At the first stage, the strata are field survey points representing six categories of place of residence, at the second-flats. The sampling frame is based on updated registers from the national census. The method of generalization includes the probability of choosing the household based on the two-stage stratified scheme described above and census-derived data on the household structure by size (post-stratification by multiplication of two weights). The standard error estimation utilized the balanced half-samples (BRR) method. For detailed information see [42]. 

The sample in the ‘Participation…’ survey was 4689 households and 12,405 individuals, of which 4642 and 12,183 (respectively) answered all the questions. Two out of three interviews were carried out with the surveyed respondent, while for one-third they were substituted by a family member due to absence or other reasons. 5504 respondents (44.4%) declared performing LTPA, either on regular or sporadic basis. These people are further referred to as physically active. Out of the extensive questionnaire, and large sample, we made use of all aspects related to table tennis that are clearly distinguishable. Thus, 346 individuals declared that they practised table tennis during the year analysed (i.e., 6.3% of physically active, and 2.8% of all respondents, unweighted). Several modules were subject to more in-depth analysis of 201 respondents (58% of all table-tennis players) who decided to include table tennis as one of five chosen disciplines and elaborated more on their practice (experience, frequency of training, cost etc.). However, as the criterion for choosing it was not clearly specified, we had to use these modules only for statistical purposes (section *Patterns of activity*). Moreover, as table-tennis rackets were put into one category with other types of rackets (tennis, badminton, squash), we refrained from expenditure estimates. The sample characteristics are presented in the Table 1. To our best knowledge, this dataset is the largest sample devoted to physical activity allowing for table tennis analysis.

In order to fulfil the purpose of this article, we measure table tennis participation and identify its determinants. For the former, we make use of descriptive statistics aimed at checking the relative and conditional frequency of table-tennis practice. In the case of large samples, the statistical significance of the percentage distribution is examined with the chi-square test with ∝ type I error and n degrees of freedom (notation: ${\chi \;}_{1-\alpha ,n}^{2})$, provided they are not weighted. 

For the latter, we utilize a binary logistic regression (binary logit model) to estimate the probability of practising table tennis within a year on the basis of demographic, socioeconomic and fitness predictors. The dependent variable equals 1 for individuals declaring that they practise table tennis within a year, and 0 otherwise (regardless of their overall physical activity). Independent variables include: gender (dummy variable), age (continuous variable, in first and second power), place of residence (categorical variable: one rural and five urban categories, depending on the number of inhabitants), natural logarithm of income per capita in a household (continuous variable, in PLN (log income per capita); the survey provides reliable and detailed information on objective income, refusals and missing values are non-existent; in the model we took the natural logarithm of the (income + 1 PLN) in order to reduce the variability of income. One PLN is added to avoid having zeros, since applying a logarithmic transformation would result in missing values then, while one PLN (approx. 0.25 EUR) is a negligible value in this context and all respondents had their incomes increased by one PLN equally), subjective financial situation of a household (categorical variable, ranging from ‘can afford some luxury’ to ‘cannot afford basic necessities’), studying (dummy variable), and number of children in a household (categorical variable, ranging from 0 to 3 and above). Due to the overrepresentation of children among table-tennis practitioners (compared to the general population), adding labour market status did not improve the overall model fit—children aged 15 and below were not asked about their labour market status. 

Despite the large sample, the fraction of table-tennis practitioners is low. This makes quantitative analysis challenging, as small fractions usually impose several restrictions on the potential methods, especially due to computational problems. As logistic regressions are sensitive to small sub-samples and fractions (lack of convergence of maximum likelihood estimations), we implement a penalized maximum likelihood estimation method proposed by Firth [43] for reducing bias in generalized linear models. The goodness of fit is examined with a Wald test (*p*-value < 0.001), a default choice for logit models, and supplemented with post-estimation classification (64.51% correctly classified respondents). We assume ∝ = 0.05. Quantitative analysis is performed with STATA 14 (StataCorp. LP, Lakeway Drive, TX, USA) with firthlogit [44] and firthfit [45] STATA modules.

## 3. Results

### 3.1. Prevalence of Table Tennis

In the last year, the involvement in table tennis was declared by 2.8% of the Polish population, which accounts for 6.6% of all physically active. It is far below the data for the most popular activities, such as cycling (66.0%), swimming (39.9%) or football (20.3%). By contrast, the combined category of tennis, badminton and squash was performed by 9.3% of all physically active. In general, individuals practising table tennis have a higher propensity to all sports categories distinguished by the questionnaire, particularly team sports (football 54.7% of table tennis players, volleyball 51.7% and basketball 36.9%), as well as tennis and related sports (41.8%). The observed increase also refers to highly saturated disciplines, such as swimming (62.8%) and cycling (81.3%), as well as seasonal activity (ice skating−increase from 8.0% to 26.5%, skiing and related−7.2% vs. 19.3%), and recreational games, such as bowling (6.5% vs. 30.0%). 

According to the model estimates (Table 2), demographic and socio-economic characteristics are statistically significant predictors of table tennis practice. Men are substantially more likely to practise the discipline—the odds of playing table tennis are over twice as high as for females, all else equal (unless stated otherwise, *p*-values < 0.001). The relationship with age is non-linear—to a first approximation, per each year the odds of practice increases, on average, by 7.9%. However, the effect is unevenly distributed between life stages—we found that it is decreasing (technically, negative and statistically significant coefficient of age squared). When comparing only the physically active, peak popularity of the discipline is noted between 10 and 19 years old (12%), between 20 and 45 it oscillates from 6 to 8% and decreases thereafter. Moreover, there is no clear age-related pattern to the gender gap (from 10.9 p.p. for men to 1.2 p.p. for women). Place of residence also visibly differentiates the likelihood of interest in the discipline—city dwellers (over 200 K inhabitants) are characterized by, ceteris paribus, 88–200% higher odds of practice than rural area dwellers, while for small town dwellers the odds do not exceed 3.5%. The categories of objective and subjective wealth provide some valuable insights: natural logarithm of per capita income (objectively measured) is statistically significant, doubling the income increases the odds of table tennis practice by roughly 13% (due to the presence of the logarithm, the interpretation of the odds ratio is not as straightforward as for other variables). The subjective financial situation indicates that poor financial conditions visibly hinder the propensity to practise table tennis—for all other categories the odds are over twice or even thrice higher, all else equal. Studying (as a pupil or student) increases the odds of practice by three times. Large families (with at least three children) are characterised by 2.39 higher odds of participating in table tennis games, compared to households without children, while for families with a child or two the odds do not rise by more than 22.5%.

### 3.2. Patterns of Activity

Among table tennis players, hedonistic motives for overall physical activity are even more frequent that in the general population (65.1% vs. 59.7%), while health issues are significantly underrepresented (7.3% vs. 13.8%). For individuals who decided to describe their table tennis practice in detail, 75% (Pearson’s ${\chi \;}_{0.95,5}^{2}$; *p* < 0.001) indicated their contact with the discipline as either occasional (e.g., during holidays) or infrequent (max. once per month), with playing time in 83% of cases not exceeding 2 h. Table tennis games are rarely organized (73% of adults and 59% of young people; 32% of school for pupils), even though the table is not basic household equipment (only 2.5% of households have one). At the same time, 56% of adult respondents have been practising table tennis for over 10 years, but the declared skills are described in 77% of cases as moderate. 

## 4. Discussion

Scientific literature devotes a lot of attention to professional table tennis, in particular, to features affecting players’ performance or talent [15,16,17,30,46,47,48,49], while articles devoted to the socio-economic determinants of recreational practice are rather scarce [38,39,40]. Except for Strawiński [38], Polish literature focuses on the historical development of the discipline and its contribution to society [50,51]. Our paper make use of the consecutive waves of the CSO survey ‘Participation of Poles in sport and physical recreation’—the only one clearly analysing LTPA broken down into disciplines, including table tennis as a separate one. Due to the methodological coherence, we are able to compare results from waves 2008 and 2012; however, they should not be analysed in terms of trends, due to insufficient number of observations. 

Even considering the overall low propensity of LTPA in Poland [4], table tennis is not a popular discipline. According our calculations, only 2.8% of the Polish population (and 6.6% of physically active Poles) practised it at least once within a year. These results are far off the charts [42], but close to the 2008 shares [38]. In fact, we have no grounds to predict a retreat from table tennis, as is suggested by Mackintosh [52] for England (from 17.5 to 14.3%) or the Outdoor Foundation for the U.S. (from 6.9 to 5.6%) [53]. A low but stable share of practitioners, and—as we show further—their concentration in selected socio-economic cross-sections suggest that table tennis belongs to less popular sports 

Unsurprisingly, table tennis is most frequent among children, and loses its popularity among adults. However, this function is not monotonous. In general, table tennis is visibly more popular among men, but nor there is a clear age-related pattern to this gender gap. The male predominance is a subject of discussion, as for young Canadians the gap was statistically insignificant [54], a visible gap referred both to practice and willingness to practise table tennis among young Chinese [55], and the analysis of a preference for practice among Welsh youth indicated a noticeable male advantage [56]. Similarly, the analysis of adult Singaporeans revealed a sharp gap in favour of men [39]. Even though Polish men are keener on LTPA than women, the interpretation of results in terms of relative roles of age and cultural factors requires further investigation. 

In the light of our analysis, income is a statistically significant predictor of leisure-time table tennis practice in Poland; however, the subjective financial condition seems to have much more impact on personal decisions on whether to participate or not. The observed positive relation between income and interest in table tennis remains consistent with Kau and colleagues [39]. Even though in Poland table tennis is dominated by practice in public facilities and the discipline is extremely rarely financed by the participants (approx. 5%), one should bear in mind that individuals living in more affluent areas have better access to PA facilities [57]—which may reflect the substantial difference between cities and rural areas or towns. Moreover, as Van Hecke et al. [58] show, in significantly deprived areas even free access to tennis tables does not have an impact on their use, due to the lack of other equipment. This may contribute to the underrepresentation of both the poorest individuals and poorest communities among table tennis players.

Patterns of amateur table tennis practice among adults suggest that it is treated more as a recreational game (akin to bowling or snooker) than as a pure sports discipline. Several observations lead to this conclusion. Firstly, there is a significant overrepresentation of hedonistic motives among those considered as dominating ones in the sports practice in general (and, consequently, underrepresentation of health-related motives). Unfortunately, the questionnaire cannot assign motives to each discipline practised by an individual (which would provide direct proof). Secondly, the table tennis practice is either occasional or infrequent and short per unit (most likely among the individuals declining to choose the detailed description table tennis the contact with the discipline was even more sporadic). Table tennis competitions are rarely organized, even though they take place in sport clubs, parks, etc. Consequently, even though the majority of adult respondents indicated that they had been practising table tennis for years, this does not translate into acquired skills.

The fact that Poles consider table tennis a leisure-time sport can be an asset. Physically active Americans claim that it is necessary to experience some benefits in order to be convinced and start regular physical activity [59]. Table tennis practised in leisure time can offer a lot of pleasure, emotions and relax, and, obviously, health benefits. Being convinced that the training is pleasant can increase the likelihood of incorporating training sessions as a priority into daily routines and more and more frequent practising. As demonstrated by scientists, the knowledge about health benefits [59] is not convincing enough to persuade people to become involved in physical activity (especially those physically inactive). Unfortunately, predominantly occasional and recreational contact with table tennis is insufficient to provide health benefits.

Apart from its obvious health advantages [25,60], flexibly adjusted table tennis training may serve individuals with various contraindications to other disciplines. For example, it can be a safe and beneficial form of movement for medical patients with cardio-respiratory disorders [61]. Moreover, it develops universal attributes (such as planning skills and hand-eye coordination), as well as separation from everyday stresses due to the full engagement it requires. As individuals rarely limit themselves to one discipline only [62], table tennis could become a tool for introducing activity those with a sedentary lifestyle. However, it requires a consistent evidence-based policy: regular promotion of table tennis as a health-oriented sports discipline aimed at overall development (not only during the Olympics), implementing table tennis into corporate social responsibility (CSR) actions of companies, similar to table football and other ventures [63,64]. In the case of children, increasing the popularity of table tennis should start from teacher-led training [65]. For both groups investments in publicly available equipment would be favourable. 

The advantages of table tennis have been appreciated by local authorities in England: the Outdoor Table Tennis Initiative (OTTI) implemented in London, as well as in other cities (the PING! project), aims at restoring egalitarian sports in their natural environment. In Poland only small-scale projects are undertaken, if at all, in locations such as playgrounds [66] or holiday resorts [67]. A project similar to the English one is currently being considered within Warsaw’s participatory budgeting for 2018 [68] and explicitly refers to the recommendation of the International Table Tennis Federation (ITTF). Possibly an evaluation of this project could become a perfect moment to analyse the possibilities of scaling-up such projects. 

Our analysis provides a unique description of who practises table tennis in Poland and how. The survey we make use of is the largest quantitative database in this area representative for the Polish population. Despite these obvious advantages, we are fully aware of its limitations. First of all, the relatively low interest in sports disciplines means that less popular disciplines cannot be described precisely. Moreover, a comparison of the results of subsequent, more up-to-date, waves of this survey would be highly recommended.

## 5. Conclusions

Our study showed that 2.8% of the Polish population (and 6.6% of physically active Poles) practised the sport at least once within a year. Among adults table tennis is predominantly performed as an occasional recreational game whereas among children, it is often the part of physical education (PE) classes. Due to its sports characteristics (training flexibility, health benefits and financial availability), there is space to make use of this sport as a tool to increase LTPA of Poles, which is very low. Pleasure, relax and emotions during recreational playing table tennis can be an initial point and provide an impulse for crossing the threshold of inactivity. The most important health gains come from moving from passive to low physical activity. In order to popularize the sport, actions should first include the purchase of tables as part of CSR measures, investments in tables in public places, better teacher training to incorporate table tennis in PE curricula and promotion of table tennis in media as a health-oriented sport.

## Figures and Tables

**Table 1:** Sample characteristics.

Variable		Sample		Physically Active		Table-Tennis Practitioners		
		*n*	%	*n*	%	*n*	%	*p*-Value
		12,406		5504		346		
gender	Male	5799	46.7	2721	49.4	233	67.3	<0.001
	Female	6606	53.2	2783	50.6	113	32.7	
age group	5–14	1452	11.7	1061	19.3	94	27.2	<0.001
	15–29	2716	21.9	1632	29.7	128	37.0	
	30–44	2645	21.3	1177	21.4	84	24.3	
	45–59	2817	22.7	959	17.4	36	10.4	
	60–74	2051	16.5	570	10.4	8	2.3	
	>74	724	5.8	105	1.9	1	0.3	
place of residence	urban, >500 K	1234	9.9	642	11.7	61	17.6	<0.001
	urban, 200–499 K	989	8.0	502	9.1	42	12.1	
	urban, 100–199 K	821	6.6	384	7.0	21	6.1	
	urban, 20–99 K	1986	16.0	911	16.6	28	8.1	
	urban, <20 K	1331	10.7	640	11.6	29	8.4	
	rural	6044	48.7	2425	44.1	145	41.9	
income quantile	Q1 (bottom quantile)	2395	19.3	1033	18.8	63	18.2	0.238
	Q2	2469	19.9	982	17.8	70	20.2	
	Q3	2492	20.1	1022	18.6	60	17.3	
	Q4	2518	20.3	1124	20.4	58	16.8	
	Q5 (top quantile)	2531	20.4	1343	24.4	95	27.5	
subjective money management	can afford some luxury	110	0.9	82	1.5	9	2.6	0.067
	can afford anything without devotion	1179	9.5	664	12.1	50	14.5	
	live sparingly	6958	56.1	3216	58.4	203	58.7	
	live very sparingly	3759	30.3	1425	25.9	81	23.4	
	cannot afford for basic expenditures	399	3.2	117	2.1	3	0.9	
household size	1	872	7.0	301	5.5	18	5.2	<0.001
	2	2720	21.9	989	18.0	46	13.3	
	3	2777	22.4	1208	21.9	57	16.5	
	4	3001	24.2	1571	28.5	121	35.0	
	5+	3035	24.5	1435	26.1	104	30.1	

Notes: *p*-value—table-tennis practitioners chi-square *p*-value, K—thousand.

**Table 2:** Demographic and socioeconomic characteristics of table tennis practice according to the model estimates.

Variable		Odds Ratio	Standard Error	*p*-Value	95% Confidence Interval	
Gender	Male	2.189	0.005	<0.001	2.179	2.198
	Female	ref.				
Age	age	1.079	0.000	<0.001	1.078	1.079
	age squared	0.999	0.000	<0.001	0.999	0.999
place of residence	urban, >500 K	1.995	0.007	<0.001	1.982	2.008
	urban, 200–499 K	1.766	0.006	<0.001	1.754	1.778
	urban, 100–199 K	0.881	0.004	<0.001	0.873	0.889
	urban, 20–99 K	0.996	0.003	0.195	0.990	1.002
	urban, <20 K	1.034	0.004	<0.001	1.027	1.042
	rural	ref.				
income per capita	log income per capita (PLN)	1.196	0.003	<0.001	1.191	1.201
subjective financial situation	can afford some luxury	3.026	0.041	<0.001	2.947	3.106
	can afford anything w/o thrift	2.436	0.029	<0.001	2.381	2.494
	must save for big purchases	2.544	0.029	<0.001	2.487	2.601
	must economize a lot	2.155	0.025	<0.001	2.108	2.204
	cannot afford basic necessities	ref.				
studying	yes	2.876	0.009	<0.001	2.857	2.894
	no	ref.				
# children in household	0	ref.				
	1	1.225	0.003	<0.001	1.218	1.231
	2	1.116	0.003	<0.001	1.110	1.123
	3+	2.392	0.010	<0.001	2.373	2.411
constant		0.001	0.000	<0.001	0.001	0.001

Notes: *n* = 5414, due to refusals. Ref.—reference category, K—thousand, #—number. Post-estimation classification: correctly classified 62.91%. Wald test of parameter significance: *p*-value < 0.001.
